# Modeling dose-response relationships of the effects of fesoterodine in patients with overactive bladder

**DOI:** 10.1186/1471-2490-10-14

**Published:** 2010-08-19

**Authors:** Linda Cardozo, Vik Khullar, Ahmed El-Tahtawy, Zhonghong Guan, Bimal Malhotra, David Staskin

**Affiliations:** 1 King's College Hospital, Denmark Hill, London SE5 9RS, UK; 2 St. Mary's Hospital, Mint Wing, Norfolk Place, London W2 1PG, UK; 3 Pfizer Inc, 235 East 42nd St, New York, NY 10017, USA; 4 Tufts University School of Medicine, Caritas-St. Elizabeth's Medical Center, 736 Cambridge St, Boston, MA 02135, USA

## Abstract

**Background:**

Fesoterodine is an antimuscarinic for the treatment of overactive bladder, a syndrome of urgency, with or without urgency urinary incontinence (UUI), usually with increased daytime frequency and nocturia. Our objective was to develop predictive models to describe the dose response of fesoterodine.

**Methods:**

Data from subjects enrolled in double-blind, placebo-controlled phase II and III trials were used for developing longitudinal dose-response models.

**Results:**

The models predicted that clinically significant and near-maximum treatment effects would be seen within 3 to 4 weeks after treatment initiation. For a typical patient with 11 micturitions per 24 hours at baseline, predicted change was -1.2, -1.7, and -2.2 micturitions for placebo and fesoterodine 4 mg and 8 mg, respectively. For a typical patient with 2 UUI episodes per 24 hours at baseline, predicted change was -1.05, -1.26, and -1.43 UUI episodes for placebo and fesoterodine 4 mg and 8 mg, respectively. Increase in mean voided volume was estimated at 9.7 mL for placebo, with an additional 14.2 mL and 28.4 mL for fesoterodine 4 mg and 8 mg, respectively.

**Conclusions:**

A consistent dose response for fesoterodine was demonstrated for bladder diary endpoints in subjects with overactive bladder, a result that supports the greater efficacy seen with fesoterodine 8 mg in post hoc analyses of clinical trial data. The dose-response models can be used to predict outcomes for doses not studied or for patient subgroups underrepresented in clinical trials.

**Trial Registration:**

The phase III trials used in this analysis have been registered at ClinicalTrials.gov (NCT00220363 and NCT00138723).

## Background

Fesoterodine is an antimuscarinic indicated for the treatment of overactive bladder (OAB) with symptoms of urge urinary incontinence, urgency, and frequency [1]. The recommended starting dosage for fesoterodine is 4 mg once daily, which can be increased to 8 mg once daily depending on patient response [1]. In 2 pivotal phase III trials, fesoterodine 4 mg and 8 mg were significantly better than placebo in decreasing the number of micturitions, urgency episodes, and urgency urinary incontinence (UUI) episodes over 24 hours (*P *< 0.01 for all comparisons) [2,3]. In a post hoc analysis of pooled phase III data, improvements in UUI episodes per 24 hours, mean voided volume (MVV), treatment response, and continent days per week were significantly greater with fesoterodine 8 mg compared with fesoterodine 4 mg (*P *< 0.05 for all comparisons) [4]. In a separate pooled analysis of the phase III data, fesoterodine 8 mg was significantly better than fesoterodine 4 mg in decreasing the number of UUI episodes among subjects with moderate or severe UUI at baseline [5].

The phase III trials evaluated only 2 doses of fesoterodine. Although the post hoc analyses suggest a dose-related increase in efficacy, data from 2 doses alone do not permit a thorough assessment of the dose-response relationship of fesoterodine. A pharmacokinetic study [6] and 2 phase II trials [7,8] included 3 doses of fesoterodine (4 mg, 8 mg, and 12 mg), providing additional data for assessing the dose relationship of pharmacokinetic exposure and of clinical effects. In the pharmacokinetic study [6], in which single doses of fesoterodine were administered to healthy male volunteers, increases in maximum plasma concentration and area under the concentration-time curve from time zero to infinity were dose proportional. In the phase II studies [7,8], all 3 fesoterodine doses were efficacious in subjects with OAB, with a linear dose-related improvement seen in number of micturitions per 24 hours [8].

Although statistically significant differences were seen favoring the fesoterodine 8-mg dose over the 4-mg dose on key efficacy endpoints in the phase III trials, a dose-response relationship in the strictest sense cannot be established with fewer than 3 dose levels. Therefore, mathematical models were developed to describe quantitative and predictive dose-response relationships of the effects of fesoterodine. These models utilize individual subject-level, longitudinal data set from two Phase II and two Phase III studies. Compared with the traditional analysis of the end-of-treatment results on study-by-study basis, this model-based dose-response characterization is more comprehensive because all available subject-level data obtained at each study visit after administration of 3 different dose levels (4, 8, or 12 mg) from a combined dataset across trials were analyzed.

The value of modeling and simulation in drug development is evidenced by its acceptability by drug regulatory agencies. As part of the U.S. Food and Drug Administration (FDA) Modernization Act of 1997, lawmakers stated that "data from one adequate and well-controlled clinical investigation and confirmatory evidence (obtained prior to or after such investigation) are sufficient to establish effectiveness [9]." Modeling results may be accepted by the FDA to support or confirm efficacy [10], as illustrated by the prescribing information for gabapentin, for example, which notes that "pharmacokinetic/pharmacodynamic modeling provided confirmatory evidence of efficacy across all doses" [11].

In addition to use in drug approval, models can be used to supplement clinical trial data by predicting outcomes for scenarios not studied in those trials. Once the validity and reliability of the model have been established, values for dependent variables, such as dose response, can be calculated for any independent variable, such as time or drug concentration [9]. Modeling and simulation can thus be used to predict effects of dose over time or time for a drug to reach maximum response in a typical patient, information of value to the practicing clinician making treatment decisions.

We describe here the use of modeling and simulation to define dose-response relationships for the clinical effects of fesoterodine on key bladder diary endpoints and postvoid residual (PVR) urinary volume in patients with OAB.

## Methods

A population analysis was conducted using pooled data from two phase II [7,8] and two phase III [2,3] studies. The protocol for each study was approved by the appropriate ethics committee or institutional review board at each study site; a list of the institutional review boards is provided in **Appendix I**. All subjects provided written informed consent before study enrollment. Each study was conducted in compliance with the Declaration of Helsinki, the International Conference on Harmonisation Good Clinical Practice guidelines, and local regulations. The phase III trials used in this analysis have been registered at ClinicalTrials.gov (NCT00220363 and NCT00138723). The protocols for the phase II trials are available at ClinicalStudyResults.org (protocol numbers SP582 [A0221027] and SP668 [A0221029]).

The data set contained pooled response, demographics and covariates, time, and dosing information. Data were analyzed using nonlinear mixed-effects modeling (NONMEM software system, version VI; GloboMax LLC, Ellicott City, Maryland). Individual estimates of parameters were obtained using POSTHOC, an empirical Bayes estimation method. The random effect models sufficiently described the error distributions. In the current modeling and simulation analysis, the developed models were evaluated for goodness of fit and then subjected to posterior predictive check (PPC) model evaluation.

Longitudinal modeling was incorporated to estimate the time when peak effects would be expected after treatment initiation. Patient demographics, such as age, sex, and baseline OAB symptoms, were evaluated as covariates of treatment response. Model simulations were used to describe expected response in patient subgroups of interest, for instance, by age and sex.

### Population Model Development

Assessment of model adequacy and decisions about increasing model complexity were driven by the data and guided by goodness-of-fit criteria, including visual inspection of diagnostic scatter plots (eg, observed versus predicted concentration, residual/weighted residual versus predicted concentration or time).

### Data Assembly

Data from 2514 subjects given placebo or fesoterodine 4 mg, 8 mg, or 12 mg in 2 phase II [7,8] and 2 phase III [2,3] double-blind 8- or 12-week trials were used to develop the dose-response models. The number of patients in each study from whom data were used is shown in Table 1. Data from a tolterodine group included in one of the phase III trials [3] were not used in model development.

**Table 1:** Number of subjects by study and dose.

	Placebo	Fesoterodine 4 mg	Fesoterodine 8 mg	Fesoterodine 12 mg	Total
Phase II					
Cole, 2004 [7]	183	186	172	186	727
Nitti et al, 2006 [8]	43	44	47	39	173
Phase III					
Chapple et al, 2007 [3]	277	263	276	0	816
Nitti et al, 2007 [2]	265	266	267	0	798
Total	768	759	762	225	2514

Exploratory data analyses were performed for each endpoint. Dose-response models were established using clinical trial data for fesoterodine 4 mg, 8 mg, and 12 mg, but efficacy results are presented only for the FDA-approved doses of 4 mg and 8 mg. While the 12-mg dose is not an approved dose, the addition of data from a total of 225 patients who received the 12-mg dose (n = 225) in Phase 2 trials and provided a total 3 dose levels to strengthen confidence in the shape of the dose-response relationship.

### Efficacy and Safety Endpoints

The 3 efficacy endpoints used in the models were number of micturitions per 24 hours, number of UUI episodes per 24 hours, and MVV per micturition. For each of these endpoints, linear and nonlinear models were evaluated to describe the expected number of micturitions, number of UUI episodes, and MVV over time for a patient treated with fesoterodine, taking into account baseline values, onset of drug and placebo effect, and treatment effect. Micturition and UUI counts follow the Poisson distribution, which is a discrete probability distribution that expresses the probability of a number of events occurring in a fixed period of time if these events occur with a known average rate and independently of the time since the last event. In general, count data are best described by the Poisson distribution and have been used previously to model UUI episodes [12].

Typical anticholinergic effects like dry mouth and constipation were generally of mild to moderate severity; any dose-relationship of adverse events and associated discontinuation rates was easily apparent from their descriptive summaries, and further exploration of the tolerability data did not reveal any significant patient covariates. Therefore, these adverse events were not considered for model-based analysis. Because post void residual urine volume (PVR) can be of potential safety concern, it was selected to fully understand the dose-response relationship and patient covariates that may influence the effect of fesoterodine treatment on PVR. Given the considerable variability in subject data from the clinical trials with regard to PVR, logistic regression equations were developed to model the probability that PVR would exceed 100 mL in a patient treated with fesoterodine at any time point, rather than modeling changes in PVR over time using longitudinal data. The 100-mL threshold was selected because subjects with a baseline PVR >100 mL were excluded from the phase II and III trials; setting a higher threshold would result in a low number of subjects above the threshold. The PVR model assessed the following covariates as potential predictors of effect: number of micturitions and UUI episodes at baseline; subject age, sex, and body mass index; and baseline laboratory values.

### Model Implementation and Performance Check

Base models tested were both linear and nonlinear with respect to dose; the treatment effect over time was modeled as either additive or proportional to baseline values. The final model evaluations included comparisons of the Objective Function value between hierarchical models. The statistical significance is considered when there is a decrease in Objective Function corresponding to a chi-square distribution with α = 0.01 and degrees of freedom equal to the difference in the number of estimated parameters between any two models was used as the criteria for final model selection. Estimates for pharmacokinetic, pharmacodynamic, and covariate effects were considered fixed; intersubject and intrasubject variables were random effects. A step-wise selection of covariates was followed to select the significant covariates. The log-likelihood ratio test was used to compare the base model without any covariates to the model with an added or deleted covariate. When addition of a covariate results in a p-value less than 0.01, the covariate is significant. The addition of covariates is followed by step-wise deletion of covariates; except, the criteria for deletion if the change in p-value is not significant (p = 0.001). The process is repeated until no further covariate is deleted from the model.

When alternative models for the dose effect on PVR were tested, the model with the minimum Akaike information criterion was the square root model, but this model was only marginally better than the linear model with similar parameter estimates. Therefore, the linear model was kept as the base model, and a logit scale was used for linear effects of dose.

Model performance was assessed using a simulation-based PPC in which the models and the study design were used to generate statistics of observed responses of 1000 simulated trial replicates, taking into account model uncertainty. The model and variables derived from the observed data set should produce simulated data similar to the original observed data without bias. The PPC provides information about the performance of random effects estimates, whereas a typical diagnostic scatter plot is primarily informative for fixed effects estimates.

The PPC was used to validate model performance by confirming that observed responses were within the posterior distribution of responses from simulated trials. Parameters in the validated model were then used to predict treatment effects for fesoterodine and placebo. The models used for each variable are shown in Additional file 1.

## Results

The structural parameters of the models were well estimated, with coefficients of variation (a measure of the precision of the estimates obtained) within 15% to 20% for each efficacy endpoint and within 35% for PVR. The PPC results indicated good model performance in simulating the longitudinal data and the end-of-treatment change from baseline after treatment with fesoterodine versus placebo. The treatment-effect estimates based on the final model parameters are summarized in Table 2.

**Table 2:** Predicted change from baseline in bladder diary variables after 12 weeks of treatment.

	Placebo	Fesoterodine 4 mg	Fesoterodine 8 mg
Micturitions per 24 hours	-1.2	-1.7	-2.2
UUI episodes per 24 hours	-1.1	-1.3	-1.4
MVV, mL	9.7	14.2*	28.4*

MVV = mean voided volume; UUI = urgency urinary incontinence.

*In addition to increase seen with placebo.

### Micturitions

**Figure 1:**
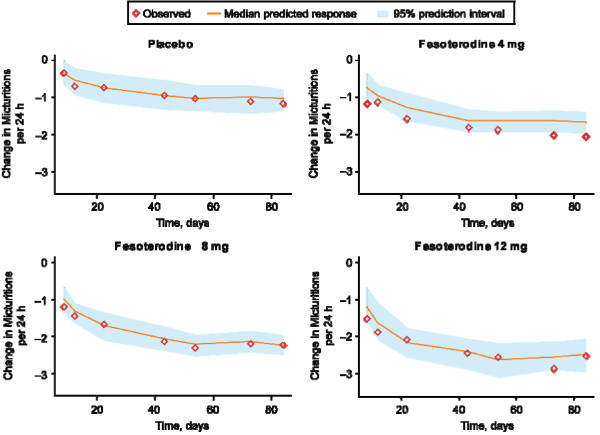
**Posterior predictive check for change from baseline in number of micturitions after taking placebo or fesoterodine 4 mg, 8 mg, or 12 mg**.

Data from 2310 subjects met criteria for inclusion in the micturition model. The exploratory data analysis of pooled clinical trial data indicated that the number of micturitions in subjects given fesoterodine decreased over time in a dose-dependent manner. The PPC for the micturition model demonstrating change from baseline over time is shown in Figure 1. For a typical patient with 11 micturitions per 24 hours at baseline treated for 12 weeks, the model predicted that the change from baseline in number of micturitions would be greater with fesoterodine 4 mg and 8 mg than with placebo (Table 2). Peak response in number of micturitions was predicted to occur within approximately 27 days, with response plateauing after 60 days.

### Urgency Urinary Incontinence

**Figure 2:**
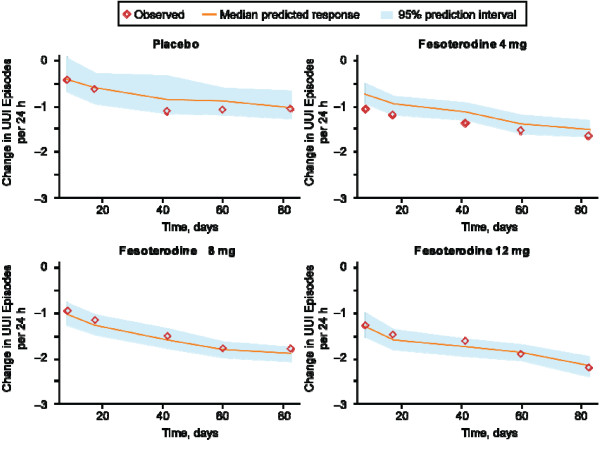
**Posterior predictive check for change from baseline in number of urgency urinary incontinence (UUI) episodes after taking placebo or fesoterodine 4 mg, 8 mg, or 12 mg**.

Data from 2505 subjects met criteria for inclusion in the UUI model. In the exploratory data analysis, the number of UUI episodes decreased over time with each fesoterodine dose. The PPC indicated that the model performed well in simulating the change in UUI episodes from baseline over time (Figure 2). For a typical patient with 2 UUI episodes per 24 hours at baseline, the predicted change from baseline after 12 weeks of treatment was greater with fesoterodine 4 mg and 8 mg than with placebo (Table 2). No plateau in response to treatment was seen, with the number of UUI episodes still decreasing at up to 80 days.

### Mean Voided Volume

**Figure 3:**
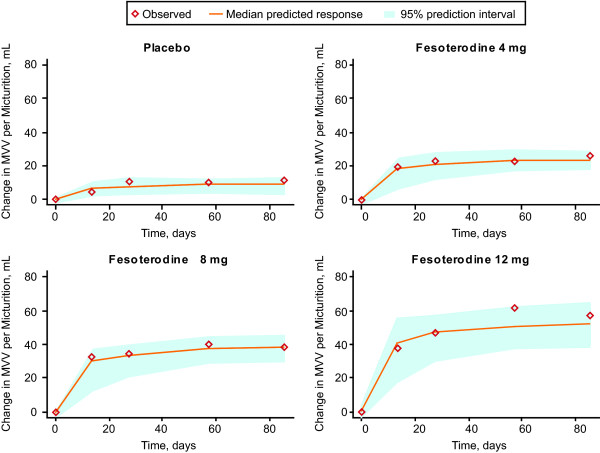
**Posterior predictive check for change from baseline in mean voided volume (MVV) after taking placebo or fesoterodine 4 mg, 8 mg, or 12 mg**.

Data from 2502 subjects met criteria for inclusion in the MVV model. The PPC showed that the model performed well in simulating change in MVV from baseline over time (Figure 3). The model predicted that, for a typical patient, the effect on MVV of 12 weeks of treatment was an estimated increase of 9.7 mL for placebo, with an additional increase of 14.2 mL and 28.4 mL for fesoterodine 4 mg and 8 mg, respectively (Table 2). Peak treatment effect on MVV was predicted to be achieved after approximately 18 days, with response over time plateauing after that.

### Postvoid Residual Volume

Data from 2385 subjects met criteria for inclusion in the final model for PVR. Overall, 161 of the 2385 subjects (6.8%) exceeded a PVR threshold of 100 mL at any point in time, 57 (2.4%) exceeded a threshold of 150 mL, and 11 (0.5%) exceeded a threshold of 200 mL. In the exploratory data analysis, PVR in general was low at baseline, ranging from 0 to 99 mL (PVR >100 mL was an exclusion criterion in the clinical trials); at last visit, PVR ranged from 0 to 404 mL. No pattern was seen in the longitudinal profile for changes in PVR, even among subjects in whom PVR reached high values at some point during the trial. The exploratory data analysis indicated that the percentage of subjects in whom PVR >100 mL was higher among men and older subjects.

**Figure 4:**
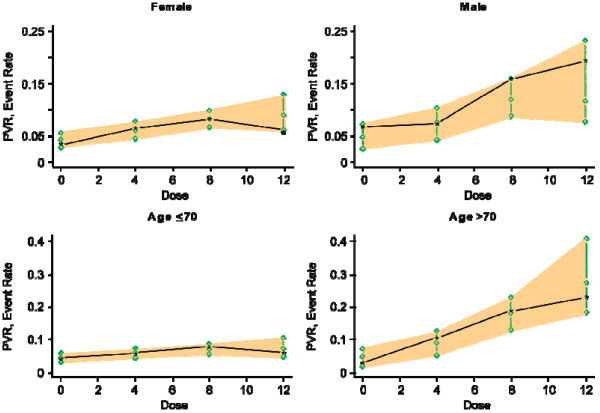
**Posterior predictive check (PPC) for the probability of postvoid residual (PVR) urinary volume >100 mL at any time point for women and men and patients aged ≤70 years or >70 years**. Circles indicate 10%, 50%, and 90% quantiles per dose; asterisks are observed rates per 1000 simulations.

In the PVR model, baseline PVR level, followed by drug dose, was the most important covariate for the probability that a patient treated for 12 weeks would develop PVR >100 mL. The PPC results indicated that the PVR model performed well in simulating the relationship between drug dose and probability that PVR would exceed 100 mL. When the PPC results were modeled by subjects' sex and age, dose dependence of the probability of PVR >100 mL was apparent in men and in older patients (aged >70 years), but there was little effect in women or patients aged ≤70 years (Figure 4).

## Discussion

The principal goal of our work was to assess the dose-response relationship for fesoterodine, which was not possible using data from the phase III clinical trials, in which only 2 fesoterodine doses were administered. To establish a reliable dose-response relationship, data from more than 2 doses are needed for modeling purposes. By including in our models data from the 2 phase III trials and 2 phase II studies, in which 3 doses of fesoterodine were used, the dose response could be estimated. The 12-mg dose data, along with data from the approved 4-mg and 8-mg doses, were used to anchor the models, particularly in evaluating nonlinear models, and to establish the appropriateness of linear models. This model-based approach for defining the dose response allowed a comprehensive evaluation of individual subject-level data collected at each visit across 4 trials encompassing placebo and fesoterodine doses of 4, 8, and 12 mg.

The final models predicted a linear dose-response relationship for fesoterodine with regard to number of micturitions, number of UUI episodes, and increase in MVV and a nonlinear pattern for treatment response over time. The values reported in table 2 represent the final model parameter estimates. The final model evaluations included Stepwise forward or backward comparisons. The selection is based on the likelihood ratio test, across multiple models, each expressing different covariate-parameter combinations [13]. The statistical significance is considered when there is a decrease in likelihood corresponding to a chi-square distribution with α = 0.01 and degrees of freedom equal to the difference in the number of estimated parameters between any two models. These findings are consistent with the results from post hoc analyses of the phase III data, in which the fesoterodine 8-mg dose was significantly more effective than the 4-mg dose for several bladder diary variables, including number of UUI episodes [4,5]

The PPC for each efficacy endpoint indicated good model performance, with the distribution of the median predicted values centered on the observed change from baseline, indicating unbiased predictions [14]. Overall, the models predicted that clinically significant effects would be seen within 3 to 4 weeks after initiation of treatment. Of note, predicted peak response in number of micturitions and MVV was seen within about 3 to 4 weeks after treatment was started, with response plateauing thereafter, but number of UUI episodes continued to decrease even after 11 weeks of treatment.

The predicted change in number of micturitions is consistent with results from the 2 phase III trials. In those studies, the least squares mean change in micturitions per 24 hours was -1.8 and -1.6 for fesoterodine 4 mg and -1.9 and -2.1 for fesoterodine 8 mg [2,3]. Modeling predicted a decrease of -1.7 and -2.2 with fesoterodine 4 mg and 8 mg, respectively. For UUI episodes, the model predictions were slightly lower than those obtained in phase III trials. The least squares mean change from baseline in UUI episodes per 24 hours in the clinical trials was -2.0 and -1.7 for fesoterodine 4 mg and -2.2 and -2.3 for fesoterodine 8 mg [2,3], compared with predictions of -1.3 and -1.4 for fesoterodine 4 mg and 8 mg in the models. For MVV, predicted results (increase from baseline of 23.9 mL and 38.1 mL for fesoterodine 4 mg and 8 mg, respectively) showed some similarity to those obtained in the phase III trials, in which the least squares mean change for MVV was 27.2 and 16.5 mL for fesoterodine 4 mg and 33.6 mL in both studies for fesoterodine 8 mg [2,3].

A dose-dependent increase in PVR >100 mL was apparent in men and patients aged >70 years but not in women or patients aged ≤70 years. The longitudinal profile of PVR did not show any relevant pattern, even in subjects who reached PVR >100 mL at some point during treatment. The risk of acute urinary retention may be related to increases in PVR. However, there is currently no threshold of PVR volume or relative increase in PVR that has been established as being associated with increased risk of AUR; 100 mL is a very conservative threshold, the clinical significance of which is unclear [15,16]. The proportion of patients with PVR >150 mL was 2.4%, and only 0.5% of patients exceeded the PVR threshold of >200 mL. For men starting treatment with an antimuscarinic, the risk of acute urinary retention, appears to be greatest during the first 30 days of treatment and particularly in the first 2 weeks [17]. Consequently, as with other antimuscarinics, it may be prudent to monitor PVR in older men starting treatment with fesoterodine, at least during the first month of treatment.

The underlying clinical data for modeling fesoterodine response were derived from trials in which subjects were randomly assigned to a fixed-dose group, which differs from clinical practice where patients can modify their dosing regimen to target a desired level of treatment response. While it might be perceived as a limitation of these analyses of fixed-dose studies as flexible-dose studies may be more clinically relevant, the latter are not suitable to elicit a pharmacologic or clinical dose response, which is best illustrated through fixed-dose studies. A broader dose range could not be explored in these models as the clinical experience with fesoterodine, based on its benefit-risk assessment, was limited to the 4-12 mg dose range evaluated in Phase II trials and 4-8 mg in Phase III trials. The fewer numbers of subjects at the 12 mg dose level are reflected in the somewhat wider confidence interval widths around the model-predicted response at that dose, particularly for micturitions and PVR. Finally, the modeling included data from patients with demography typically represented in OAB clinical trials (about 80% females and average age 65 yrs); nevertheless the covariate analysis in these models helps predict the population under-represented in the trials.

Evaluating the dose-response relationship of new drugs is necessary to establish which doses will provide the greatest efficacy with the fewest adverse effects. If the dose-response relationship is linear, then each increase in dose yields an increase in effect, whether positive (improved efficacy) or negative (worsened tolerability or safety), or both. Dose-response data can be obtained from clinical trials, but inclusion of a large number of endpoints, such as multiple dosing regimens, is not appropriate for trial subjects and may not be feasible for investigators or sponsors. Model-based drug development provides a supplemental means of assessing the risks and benefits of increasing drug dosage. Although development of valid models can be complex and time consuming, modeling offers a reasonable and valuable approach to predicting the effects of increasing dose on clinical endpoints, information that may be useful in clinical practice.

In addition to supplementing or confirming data obtained in clinical trials, models can be used to simulate outcomes using variables not included in preclinical or clinical studies [14]. These simulations can be used to predict the probability of an event and to evaluate the effects of covariates, such as subject age and sex; for instance, in our work, models were used to assess the probability of increased PVR in subgroups specifically stratified by age and sex.

## Conclusions

The results from our models demonstrate the value of modeling for increased understanding of the benefits and risks of modifying the dose of fesoterodine in treating patients with OAB. The modeling and simulation results quantitatively demonstrate a dose-response relationship for the effect of fesoterodine on key clinical OAB endpoints.

## Competing interests

This study was sponsored by Pfizer Inc. LC has received funding for research, lecturing, or consultancies from Astellas, Pfizer, and Schering-Plough and has done research consultancy or advisory work for Astellas, Pfizer, Schering-Plough, Allergan, Rottapharm, and SPE Pharma. VK is a consultant/investigator for Allergan, Astellas, Bioxell, Novartis, and Pfizer Inc. DS has received grant support and served as consultant and speaker for Pfizer, Allergan, Astellas, and Watson. AE-T, ZG, and BM are employees of Pfizer Inc.

## Authors' contributions

Concept: LC, VK, AE-T, ZG, BM, DS; Model design/generation of data: AE-T and BM; Interpretation of data: LC, VK, AE-T, ZG, BM, DS; Drafting/revising manuscript and approval of publication: LC, VK, AE-T, ZG, BM, DS.

## Appendix I: Institutional Review Boards

Aleksander Dubrzynski, MD Komisja Etyczna przy Akademii Medycznej, Warsaw, Poland

AM w Lublinie, Lublin, Poland

Andrzej Chilarski, MD Komisja Etyki Badañ Naukowych, Lodzi, Poland

Berlin, Germany

Board of Research Associates, New York, NY, USA

Bro Taf Health Authority, Cardiff, UK

Cambridge Local Research Ethics Committee, Cambridge, UK

Canterbury Ethics Committee, Christchurch, New Zealand

Carolinas Healthcare System IRB, Charlotte, North Carolina, USA

CCPPRB d'Aulnay-sous-Bois, Service Pharmacie, Aulnay-Sous-Bois, France

Central Ethics Commission, Department of Quality Control Ministry of Health, Moscow, Russia

Clinical Research Ethics Committee, Victoria, Australia

Comitato Etico Fondazione Centro San Raffaele del Monte Tabor, Milano, Italy

Comitato Etico Indipendente del Policlinico Universitario di Udine, Udine, Italy

Comitato Etico per la Sperimentazione dell'A.O, Verona, Italy

Comité Ético de Investigación Clínica des Illes Balears, Illes Balears, Spain

Comité Ético de Investigación Clínica, Alicante, Spain

Comité Ético de Investigación Clínica, Madrid, Spain

Comité Ético de Investigación Clínica, Santa Cruz de Tenerife, Spain

Comité Ético Fundación Hospital de Alcorcón, Madrid, Spain

Comité Ético, Pamplona, Spain

Comité voor Medische Ethiek, Leuven, Belgium

Commissie Medische Ethiek - Akademisch Hospital VUB, Brussels, Belgium

Commissie voor Medische Ethiek ZNA/OCMW Antwerpen, Antwerpen, Belgium

Commission d'Ethique Brussels, Belgium

Community Memorial Hospital, Menomonee Falls, Wisconsin, USA

Concord Hospital Drug Committee & CRGH Human Research Ethics Committee, Australia

Copernicus Group IRB, Cary, NC, USA

Crescent City Institutional Review Board, New Orleans, LA, USA

Department of Clinical Investigation, Madigan Army Medical Center, Tacoma, WA, USA

Ethical Committee AZ St. Dimpna, Geel, Belgium

Ethics Committee of Fovársi Önkormányzat Bajcsy-Zsilinszky Hospital, Budapest, Hungary

Ethics Committee of Grof Eszterhazy Hospital, Papa, Hungary

Ethics Committee of the State Pharmacological Center of the Ministry of Healthcare, Kiev, Ukraine

Ethics Committee University Hospital Antwerpen, Edegem, Belgium

Ethics Committee within the Federal Department for Control over Quality, Efficacy and Safety of Pharmaceutical Products, Moscow, Russia

Ethics Committee, University of Cologne, Köln, Germany

Ethikkommission der Ärztekammer Berlin, Berlin, Germany

Ethikkommission der Ärztekammer Hamburg, Hamburg, Germany

Ethikkommission der Ärztekammer Nordrhein, Düsseldorf, Germany

Ethikkommission der Landesärztekammer Baden-Württemberg, Stuttgart, Germany

Ethik-Kommission des Universitätsklinikums Charité der Humbold-Universität zu Berlin,

Ethisch Comité Heymans instituut UZ, Gent, Belgium

Eticka komise, Brno, Czech Republic

Eticka komise, Chomutov, Czech Republic

Eticka komise, Liberec, Czech Republic

Eticka komise, Olomouc, Czech Republic

Eticka komise, Plzen, Czech Republic

Etická komise, Praha, Czech Republic

Etická komise, Ústí nad Labem, Czech Republic

Evanston Northwestern Healthcare Research Institute Institutional Review Board, Evanston, IL, USA

Ewa Marcinowska-Suchowierska, MD Komisja.Bioetyczna, Warzawa, Poland

Forskningsetikskommittén Avd. Göteborg, Sweden

Forskningsetikskommittén Uppsala Universitet, Uppsala, Sweden

Graduate Hospital Institutional Review Board, Philadelphia, PA, USA

Greenville Hospital System Institutional Review Committee, Greenville, SC, USA

HCI Local Research Ethics Committee, Glasgow, UK

Health Sciences Committee Vanderbilt University Medical Center, Nashville, Tennessee, USA

Helsinki Committee, Beer Yaakov, Israel

Helsinki Committee, Hadera, Israel

Helsinki Committee, Haifa, Israel

Helsinki Committee, Jerusalem, Israel

Helsinki Committee, Rehovot, Israel

Helsinki Committee, Tel Aviv, Israel

Hôpital Robert Ballanger, Centre Daniel Eisenmann, Aulnay Sous Bois Cedex, France

Human Research Ethics Committee & Medical and Allied & Clinical Drugs Trials Committee, Nedlands, Western Australia, Australia

Huntingdon REC, Cambridge, UK

Jerzy Umiastowski, MD Komisja Bioetyczna przy, Gdynia, Poland

Karolinska Institutets Regionala Forskningsetikkommitté, Stockholm, Sweden

Komisja Bioetyczna przy Okręgowej Radzie Lekarskiej Wielkopolskiej Izby, Poznań, Poland

Komisja Bioetyczna, Szczecin, Poland

Local Ethics Committee of Fovársi Önkormányzat Uzsoki Utcai Hospital, Budapest, Hungary

LREC, London, UK

Medical Research Council Ethics Committee for Clinical Pharmacology, Budapest, Hungary

METC AZM, Maastricht, Netherlands

Ministry of Public Health of Ukraine Ethics Committee, Kiev, Ukraine

Niezalez.Komisja Etyki, Gdansk, Poland

Norwich District Ethics Committee, Norwich, UK

NYUSM IRB, New York, New York, USA

Okręgowa Izba Lekarska w Krakowie, Kraków, Poland

Okręgowa Izba Lekarska, Białystok, Poland

Pharma Ethics Ltd., Irene Republic of South Africa

Plymouth LREC, Plymouth, UK

Princess Alexandra Hospital Research Ethics Committee, Queensland, Australia

Redcliffe-Caboolture Health Service District Ethics Committee, Redcliffe, Queensland, Australia

Regional Research and Ethics Committee of Medical Scientific Council, Györ, Hungary

Regional Scienctific and Research Ethics Committee, Budapest, Hungary

Regionala etikprövningsnämnden BMC, Uppsala, Sweden

Repatriation General Hospital Research & Ethics Committee, Adelaide, South Australia, Australia

Royal Brisbane Hospital & Women's Hospital Health Services District, Office of the Human Research Ethics Committee Herston Road, Queensland, Australia

Schulman Associates IRB, Cincinnati, Ohio, USA

Scientific Ethics Committee for Copenhagen's Country, Glostrup, Denmark

Slaska Izba Lekarskiej, Katowice, Poland

South Birmingham Local Research Ethics Committee, Birmingham, UK

South Cheshire Local Research Ethics Committee, Chester, UK

South Eastern Sydney Area Health Service Research Ethics Committee, New South Wales, Australia

South Manchester Research Ethics Committee, Manchester, UK

South Sheffield Research Ethics Committee, Sheffield, UK

South-East Multi-centre Research Ethic Committee, London, UK

Southern Health Human Research & Ethics Committee, Victoria, Australia

Southmead Local Research Ethics Committee, Bristol, UK

Specialized Committee for Approving Clinical Trials of Drugs, Sofia, Bulgaria

St. Petersburg Pavlov Medical University, St. Petersburg, Russia

Tallinn Medical Research Ethics Committee, Tallinn, Estonia

The Committee on Clinical Investigators, New Procedures and New Forms of

Therapy, Boston, MA, USA

The National Ethics Committee for Clinical Trials of Medicinal Products, Bucharest, Romania

University of California, Los Angeles Medical Institutional Review Board, Los Angeles, CA, USA

University of Witwatersrand Human Research Ethics Committee, Gauteng, South Africa

West Berkshire Local Research Ethics Committee, Reading, UK

Western Sydney Area Health Service Research Office Clinical Sciences, Westmead, New South Wales, Australia

## Pre-publication history

The pre-publication history for this paper can be accessed here:

http://www.biomedcentral.com/1471-2490/10/14/prepub

## Supplementary Material

Additional file 1 **Appendix II**. The models used for each variable Click here for file
