# Supplementary material for: Modeling dose-response relationships of the effects of fesoterodine in patients with overactive bladder
Source: BMC Urol. 2010 Aug 19;10:14. doi: 10.1186/1471-2490-10-14 (PMC2939595; doi:10.1186/1471-2490-10-14)
Supplement: Additional file 1 — Appendix II. The models used for each variable [file 1471-2490-10-14-S1.DOC]

| **Variable** | **Model** |
| --- | --- |
| Micturitions | The micturition count follows a Poisson distribution and the expected number of micturitions for patient i at time j, λ_ij_, depends on baseline, time, and treatment as follows: $\begin{matrix} mict_{ij}\xrightarrow{}Poisson\left(\lambda_{ij}\right) \\ \lambda_{ij}=\exp\left[\log\left(bsl_{i}\right)\cdot\left(1-Eff_{ij}\right)+\eta_{i}\right] \\ Eff_{ij}\left(dose,t_{ij}\right)=\left(Plac+Deff\cdot dose\right)\cdot\left[1-\exp\left(-K\cdot t_{ij}\right)\right] \end{matrix}$   where bsl_i_ is the averaged observed baseline count for patient i, Eff_ij_ is treatment effect for the i^th^ patient at time j, Plac is the maximum placebo effect (at time infinity), Deff is the drug effect linearly dependent on drug dose, K is a first-order rate constant for the onset of drug and placebo effect with time, and η_i_ is a random effect for treatment in patient i that follows a normal distribution with mean 0 and variance Var(η). |
| UUI | The UUI episode count was modeled as a Poisson random variable as described for micturitions. The expected number of micturitions for patient i at time j, λ_ij_, depends on baseline, time, and treatment as follows: $\lambda_{ij}=\exp\left[\log\left(bsl_{i}\right)+\left(Plac+Deff\cdot dose\right)\cdot t_{ij}^{\gamma}+\eta_{i}\right]$   where bsl_i_ is the averaged observed baseline count for patient i, Plac is the placebo effect, Deff is the drug effect linearly dependent on drug dose, γ describes the nonlinearity of the onset of drug and placebo effect with time, and η_i_ is a random effect for treatment in patient i that follows normal distributions with mean 0 and variances Var(η). |
| MVV | MVV was modeled as a function of baseline, time, and treatment as follows: $MVV_{ij}=bsl_{i}+\text{ }(Plac\text{ }+\text{ De}ff\cdot dose + \eta_{i}) (1-e^{-K.time_{j}})\text{ +}\varepsilon_{\text{ij}}$   where MVV_ij_ is MVV for the i^th^ patient at time j, bsl_i_ is MVV observed at baseline for patient i, Plac is the maximum placebo effect (at time infinity), Deff is the drug effect linearly dependent on drug dose, K is a first-order rate constant for the onset of drug effect with time, η_i_ is a random effect for treatment in patient i, and ε_ij_ is the residual error for patient j at time i. Random effects follow normal distributions with mean 0 and variances Var(η) and $\sigma^{2}$, respectively. |
| PVR | Owing to the highly variable patterns of the individual profiles it was decided to model the probability to exceed a PVR threshold T (T=100 ml) at any point in time rather than modeling the time course of the longitudinal data.  The threshold of 100 mL was selected because subjects were not permitted to enter the studies with a baseline PVR ≥100 mL or more for medical reasons, and setting a higher threshold would result in a low fraction of patients above the threshold.  A logistic regression model was developed where the probability of exceeding a PVR threshold: T is modeled as a function of dose and covariates.  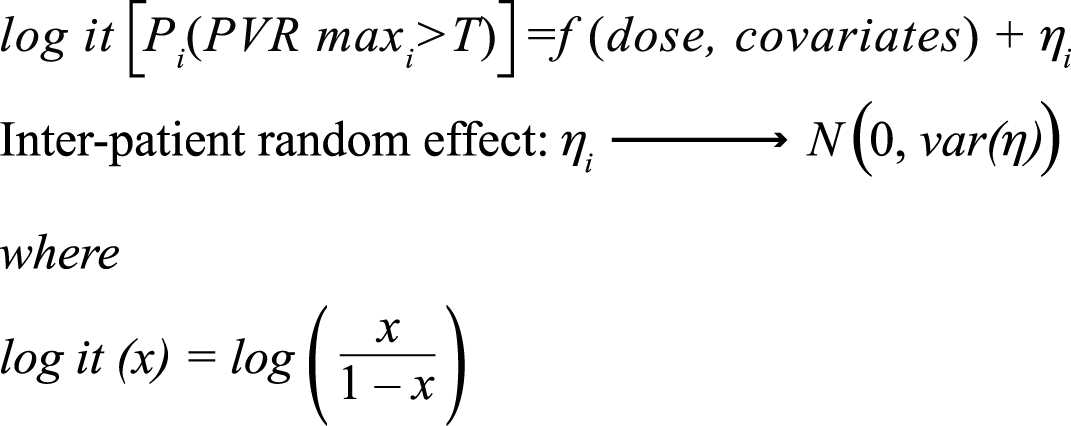 |
| MVV=mean voided volume; PVR=postvoid residual volume; UUI=urgency urinary incontinence. | |
